# Treadmill Exercise Buffers Behavioral Alterations Related to Ethanol Binge-Drinking in Adolescent Mice

**DOI:** 10.3390/brainsci10090576

**Published:** 2020-08-20

**Authors:** Patricia Sampedro-Piquero, Carmelo Millón, Román D. Moreno-Fernández, María García-Fernández, Zaida Diaz-Cabiale, Luis Javier Santin

**Affiliations:** 1 Departamento de Psicobiología y Metodología de las Ciencias del Comportamiento, Facultad de Psicología, Universidad de Málaga, 29071 Málaga, Spain; roman.moreno@uma.es; 2 Instituto de Investigación Biomédica de Málaga (IBIMA), 29010 Málaga, Spain; carmelomp@uma.es (C.M.); igf@uma.es (M.G.-F.); zaida@uma.es (Z.D.-C.); 3 Departamento de Fisiología Humana, Facultad de Medicina, Universidad de Málaga, 29071 Málaga, Spain

**Keywords:** adolescence, aerobic exercise, alcohol, behavior, drinking in the dark, mice

## Abstract

The binge-drinking pattern of EtOH consumption, which is frequently observed in adolescents, is known to induce several neurobehavioral alterations, but protection strategies against these impairments remain scarcely explored. We aimed to study the protective role of treadmill physical exercise on the deficits caused after repeated cycles of binge-like EtOH exposure in the cognition, motivation, exploration, and emotion of C57BL/6J mice from adolescence to adulthood. Animals were divided into four groups: control group, exercised group, EtOH group, and exercised + EtOH group (20% in tap water). The exercise was performed for 20 min, 5 days/week at 20 cm/s. Then, animals were submitted to several behavioral tasks. Compared to binge-drinking mice, the exercised + EtOH group exhibited diminished anxiolytic-related behaviors in the elevated plus-maze, enhanced exploratory activity in the open field, reduced preference for alcohol odor when another rewarding stimulus was present (social stimulus) and lower latency to start self-cleaning behaviors in the sucrose splash test. In contrast, other measurements such as habituation learning and working memory were not improved by exercise. Besides, exercise was not able to reduce alcohol consumption across the weeks. In conclusion, physical activity during adolescence and early adulthood could buffer certain neurobehavioral alterations associated with binge-drinking, despite not reducing the quantity of consumed alcohol.

## 1. Introduction

Binge drinking (BD) is a dangerous and emerging pattern of alcohol consumption characterized by repeated short episodes of heavy intake followed by detoxification and extended periods of abstinence [1,2]. It is frequently observed in adolescents and young adults whose brain, not yet mature, is very susceptible to the detrimental effects of alcohol [3,4]. Several preclinical and clinical studies have revealed that BD is associated with long-term behavioral and neurobiological impairments along with a risk of dependence in adulthood [5,6].

Owing to this, some preclinical studies have tried to overcome the long-term consequences of alcohol exposure being environmental enrichment (EE) one of the most applied treatments [7,8]. Enriched rodents are typically housed in large groups and exposed to a variety of stimuli that can provide both cognitive stimulation and physical exercise [9]. Several studies have revealed that physical exercise is a critical component of this intervention because exercise alone has shown to produce many of the benefits of EE [10,11]. Physical activity promotes neuroplasticity in certain brain structures leading to cognitive improvements, a reduction of anxiety-related behaviors, better coping strategies, and enhancement of motivation and well-being [12,13,14,15]. Hence, exercise is increasingly being used as an adjunctive treatment for alcohol use disorders in humans [16,17], and research using animal models has revealed that physical activity can reverse binge-induced brain damage and promote neuroprotection [18,19]. Moreover, it is possible that the influence of exercise on the brain during adolescence could act as a mainstay for future neural and behavioral outcomes [20]. Nevertheless, few studies have analyzed the potential for physical activity as a protection strategy, and the few that have focused on this issue have only assessed its capacity to reduce or avoid brain alterations, but they have not studied its effect on behavioral and cognitive alterations involved in EtOH BD [21,22].

Therefore, although the negative impact of EtOH BD during adolescence on behavior and cognition in later life is well-known, few studies have been conducted on protective strategies. Hence, we aim to assess the effect of a moderate treadmill exercise protocol concurring with binge alcohol consumption on several behavioral domains (cognitive, anxiety, exploration, motivation) in adolescent male mice. Ethanol consumption was carried out in mice from pnd32 to pnd63 which corresponds to early adolescence and early adulthood, respectively. Nevertheless, it is debatable whether a mouse at pnd 55–65 should be considered a young adult or a late-adolescent [23]. In human terms, these postnatal days refer to puberty and early adulthood. The average age at which mice attain puberty is approximately 42 days (pnd42) and the average age in humans is approximately 11.5 years [24]. Thus, in this phase, one human year is equivalent to 3.65 mice days [25].

## 2. Materials and Methods

### 2.1. Animals

A total of 38 male C57BL/6J mice were acquired from the Janvier Labs (Le Genest-Saint-Isle, France) at 21 days old and underwent one week of acclimatization before the experiment started. Animals were group-housed (21.5 × 46.5 × 14.5 cm) in standard conditions (temperature: 22 °C ± 2 °C; humidity: 60 + 10%) under a 12 h light-dark inversed cycle (red light on at 9:00 h). *Ad libitum* food and water were available for the animals except during alcohol access. Mice were randomly assigned to four groups: a control group (CON, n = 9), an exercised group (EX, n = 9), an EtOH group (EtOH, n = 11), and an exercised + EtOH group (EX + EtOH, n = 9). Procedures followed the European Directive 2010/63/UE; 90/219/CEE, Regulation EC No. 1946/2003) and Spanish regulations (Real Decreto 53/2013 and Ley 32/2007) for animal research. The experimental protocols were approved by the research ethics committee at the University of Malaga (CEUMA 24-2018-H).

### 2.2. Procedure

#### 2.2.1. Treadmill Exercise

The exercise training was performed using a single-lane treadmill for mice (Harvard Bioscience, Inc., Panlab, Barcelona, Spain). Exercise was performed 5 days/week at a speed of 20 cm/s with no inclination of the treadmill during postnatal day (pnd) 32 to 63. Previously, animals were familiarized with the treadmill apparatus for 3 days to minimize novelty stress [26]. During the third day, the duration was increased to 20 min, with a speed of 20 cm/s. If the animals refused to run, a gentle tail touch with a small stick was used to motivate the mice to run. Mice from the EX + EtOH group were trained between 9:00 and 12:00 before access to EtOH and the EX group was trained between 12:30 to 15:30.

#### 2.2.2. Drinking in the Dark Procedure (DID)

Animals from the EX + EtOH and EtOH groups were exposed to alcohol from pnd 32 to 63. Each week, mice were exposed for 2 h (12:00 to 14:00, 3 h after lights off) to a single bottle containing an EtOH solution (20% in tap water) for three consecutive days and for a 4 h session on the fourth day (12:00 to 16:00). In the following three days, animals had no access to alcohol [27]. The EtOH solution was prepared from 100% EtOH in tap water, and the total solution available for the mice was 10 mL. During the drinking sessions, animals were singly housed (identified by ear punch) in similar plastic cages and control animals were submitted to the same manipulation (CON and EX groups experienced the same isolation and food restriction), although in their cages the single bottle contained tap water (10 mL). Water and EtOH solutions were replenished daily. Alcohol consumption was calculated daily by weighing bottles before and after exposure to drinking. Body weight was also controlled during the DID protocol. Additional bottles with the EtOH solution were included to control for spillage and evaporation and at the end of the DID session, we calculated the difference between their initial and final weights and subtracted the average value from the other bottles.

The animals belonging to the EX + EtOH were single housed at 11:00 h, one hour before starting the DID session. However, because we only had a single-lane treadmill, we had to train the animals one at a time and not all finished the training by 11 h. Thus, in the EX + EtOH group, which comprised 9 animals, 3 had yet to be trained by that point, so to avoid possible effects on the DID session we randomized the order of training in this group every day. The EtOH, EX, and the CON groups were also single housed one at a time from 11 h in the same way as the EX + EtOH group. The isolated conditions were maintained from 11 h to 14 h or from 11:00 h to 16:00 h, depending on the DID session (2 h or 4 h), for all animals. DID water sessions for the EX and the CON groups were performed during the same time period as the ethanol sessions (12–14 h and 12–16 h). In the case of the EX group, the animals performed the 20 min of exercise in random order each day and were returned to their boxes until the DID session had ended. When the DID session finished, all the animals were returned to group conditions.

#### 2.2.3. Blood EtOH Concentration (BEC)

Blood samples were collected from the tail veins after the 4 h sessions on the first, intermediate, and last week of alcohol exposure. Animals from CON and EX groups were immobilized during the same time by the experimenter to expose them to similar stressful experimental conditions. Blood samples were collected in capillary tubes that contained EDTA dipotassium salt (Microvette^®^ 100 µL, Sarstedt, Nümbrecht, Germany). Blood was centrifuged at 6000 rpm (Heraeus Biofuge Fresco, Hanau, Germany) for 10 min at 4 °C, and the plasma was stored at −80 °C. BEC was determined using a commercial EtOH assay kit following the protocol recommended by the manufacturer (Abcam, Cambridge, UK, Ethanol Assay Kit, ab65343).

#### 2.2.4. Behavioral Assessment

**Figure 1:**
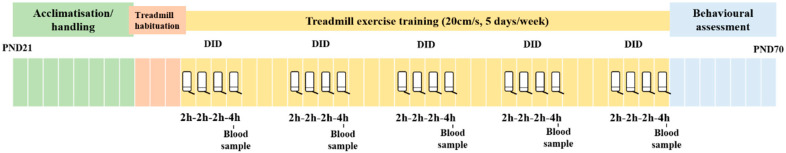
Schematic representation of the experimental timeline. C57BL/6J mice were exposed to a 5-week Drinking the Dark (DID) procedure. Every 15 days after a 4 h session, a blood sample was obtained from the lateral tail vein to analyze BECs. Concurrently, animals from the EX and EX + EtOH groups were submitted to treadmill exercise training (20 cm/s) for 20 min, 5 days/week. Finally, from pnd 64 to 70, all animals were tested on different behavioral tasks.

From days 64–70, a behavioral battery of tests was employed to assess exploratory activity, anxiety-related behaviors, stress-coping strategies, and motivational and cognitive domains. The behavioral assessment was performed in the following sequence: the elevated plus-maze test (EPM, Day 64), the Y-maze test (Day 65), the open-field test (Day 66, 67), the nest building test (Day 68), the sucrose splash-test (Day 69), and the three-chamber test (Day 70). Behavioral variables were analyzed automatically, some of them manually, with the software Ethovision XT 12 (Noldus, Wageningen, The Netherlands). After each session, the apparatuses were carefully cleaned with a solution of 70% alcohol to remove the odor cues. Behavioral testing was carried out during the dark phase of the animals between 10:00 h to 14:00 h. Details about the apparatuses’ dimensions and characteristics are shown in the Supplementary Material section. Study design flowchart is displayed in Figure 1.

#### 2.2.5. Elevated Plus Maze

The EPM test was used to assess anxiety-like behaviors [25]. Mice were placed in the center of the apparatus and allowed to explore for 5 min. The behaviors analyzed with the Ethovision XT 12 software included locomotion (cm), time spent in open arms (s), latency to enter into a closed arm (s), time spent in the center (s) and percentage of open arm entries ((open arm entries/total arm entries) * 100).

#### 2.2.6. Y Maze

The Y-maze test was used to measure spatial working memory [28]. Each animal was placed in the center of the Y-maze and was free to explore the arena for 8 min. An arm entry was scored when the mouse placed the four paws within that arm. The following dependent variables were registered manually: total number of arm entries, percentage of perseveration (number of repeated entries to the same arm/total of entries * 100), and percentage of alternation ((number of entries into three different arms on consecutive choices/maximum number of possible alternations minus 2) * 100). An alternation was defined as an entry into three different arms on consecutive choices.

#### 2.2.7. Open Field Test

The Open-field test was used to assess exploratory activity [28], as well as habituation learning performing two sessions separated by 24 h. Mice were placed in the center of the maze, and they were allowed to explore a squared open field for 10 min. Distance traveled (cm), velocity (cm/s), latency to enter into the periphery, and time spent in the center area were automatically analyzed, whereas the latency to enter into the center, rearing and grooming were manually registered. As the animals started the trial in the center of the maze, we defined this last variable as the time spent between the mice going to the periphery and returning to the central area of the field.

#### 2.2.8. Nest Building Test

In mice, nest building is an ethological and relevant behavior involved in heat maintenance, reproduction, and shelter [29]. Mice were individually housed, and they were provided with two Nestlets (a 5 cm square of compressed cotton-like material weighing approximately 2.4 g; purchased from Ancare, Bellmore, NY, USA), which were placed in the cage at 10:00. At 1 h and 24 h later, the quality of the nest built was evaluated by a researcher trained and blind to the experimental conditions [30]. The scoring system for assessing nest quality was carried out based on Deacon’s standardized scale from 1 to 5.

#### 2.2.9. Sucrose Splash Test

This test was employed as an index of self-care and motivational behavior. Mice were singly housed and vaporized with a 10% sucrose solution on their back fur. This caused the mice to initiate grooming in response to the solution viscosity. The number of grooming responses (head or body grooming), the latency to start this behavior, and the time spent grooming were recorded over 5 min and manually analyzed [31].

#### 2.2.10. Three-Chamber Test

This test assesses the interest in social novelty because rodents often prefer to spend more time interacting with one another [32]. In our case, testing occurred in two sessions (interval between sessions of 1 h) within a three-chambered box with openings between the chambers (Panlab, Harvard apparatus). The first session was a habituation to the empty box (5 min). During the second trial (10 min), the mice could choose to explore a never-before-met juvenile mouse (3–4 weeks old) placed in one grid enclosure or a bottle with 20% alcohol in tap water (20 mL) in the other grid enclosure. The bottle with alcohol was replaced in each animal session to maintain the odor. Animals could smell the alcohol, but not drink it. The time spent sniffing each grid enclosure (manually analyzed) and the time spent in each chamber were recorded (Ethovision XT12). To maintain the alcohol odor in the compartment where the bottle was placed, the apparatus was cleaned only with water, and the fecal boli were removed.

### 2.3. Data Analysis

Repeated-measures analysis of variance was carried out to analyze EtOH intake, BECs, and changes in body weight over time. Behavioral variables of the different tests were analyzed with two-way ANOVAs, in which exercise (exercise/unexercised) and ethanol (ethanol/no ethanol) were the two factors with two levels each, or two-way repeated-measures ANOVAs depending on the test. These analyses were followed by an honestly-significant-difference (HSD) Tukey’s post-hoc test when significant differences were found. Pearson’s correlation was calculated between BEC levels and EtOH intake (g/kg) of the last 4 h session. Only statistically significant differences (*p* < 0.05) are displayed. Data are expressed as mean ± standard error of the mean (SEM). Graphs of the variables without significant differences are displayed in the Supplementary Material.

## 3. Results

### 3.1. EtOH Intake and BEC

**Figure 2:**
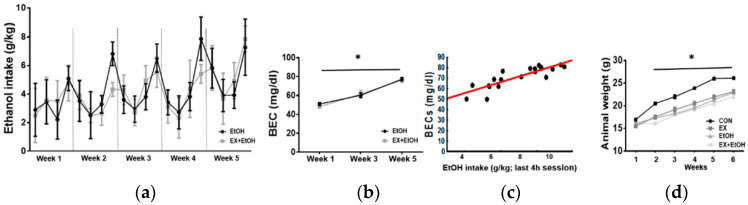
Changes in alcohol consumption and weight of the animals during DID protocol. (**a**) Significant differences were not found between groups, but significant effects of the session (*p* = 0.001) and week (*p* < 0.0001) were observed. (**b**) BEC levels during the first, third, and fifth weeks and animal weight in the different experimental conditions. BECs in the EtOH and EX + EtOH groups are expressed as milligrams per deciliter (mg/dL). The quantity of alcohol in the blood increased over time (*p* < 0.0001), but significant differences were not found between groups. (**c**) A significant correlation between BEC levels and EtOH intake in the last 4 h session was found (*p* < 0.001). (**d**) Animal weight over time. All data are mean ± SEM.

The EtOH and EX + EtOH groups consumed similar levels of alcohol (*F* (1, 76) = 2.13, *p* = 0.32). Repeated-measures ANOVA showed a significant effect of sessions (*F* (1, 76) = 34.94, *p* = 0.001) and weeks (*F* (4, 304) = 7.59, *p* < 0.0001), but interactions were not significant (*p* > 0.05) (Figure 2a). The BEC analysis revealed that the quantity of alcohol in blood increased over time (*F* (2, 36) = 15.22, *p* < 0.0001), but significant differences were not found between groups (*F* (1, 18) = 0.10, *p* = 0.98; Figure 2b) or in the interaction weeks x groups (*F* (2, 36) = 1.24, *p* = 0.30). Finally, a positive Pearson’s correlation was found between BEC levels and EtOH intake in the last 4 h session (*r* = 0.8592, *p* < 0.001; Figure 2c).

### 3.2. Body Weight

The body weight of the animals increased over time (*F* (5, 170) = 527.87, *p* < 0.0001). The factor groups showed statistical significance (*F* (3, 34) = 33.04, *p* < 0.0001), and the interaction weeks x groups was also significant (*F* (15, 170) = 7.12, *p* < 0.0001). HSD Tukey’s post-hoc test revealed that the CON group had a higher weight compared to the other groups on weeks 2, 3, 4, 5, and 6 (*p* = 0.002; Figure 2d).

### 3.3. Behavioral Results

#### 3.3.1. Emotionality and Social Preference

*EPM*

**Figure 3:**
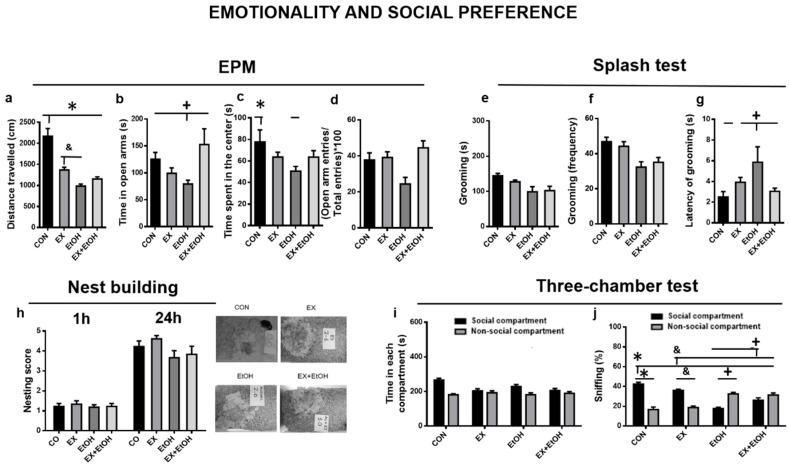
Behavioral performance in Emotionality and Social preference. (**a**–**d**: Elevated-plus maze; **e**–**g**: Sucrose splash test; **h**: The nest building test and representative photographs of nests from the different experimental conditions at 24 h; **i**,**j**: Three-chamber test). All data are mean ± SEM, and statistically significant differences were considered when *p* < 0.05. * Statistical differences between the CON group compared to other groups; + Statistical differences between the EtOH group compared to other groups; statistical differences between the EX group compared to other groups.

*A* two-way ANOVA revealed significant interactions *ethanol x exercise* in the distance traveled (*F* (1, 34) = 35.06, *p* < 0.0001; Figure 3a), time in open arms (*F* (1, 34) = 7.01, *p* = 0.01; Figure 3b) and time spent in the center (*F* (1, 34) = 3.92, *p* = 0.05; Figure 3c). Tukey’s post hoc analysis revealed that the CON group traveled a further distance than the other groups (*p* < 0.001) and spent more time in the center of the maze compared to the EtOH group (*p* = 0.03; Figure 3c). The EX group traveled further than the EtOH group (*p* = 0.04; Figure 3a). The EtOH group spent less time in the open arms (CON and EX + EtOH: *p* = 0.01; EX: *p* = 0.05). In the case of the variable (open arm entries/total entries) * 100, the main effects *ethanol* and *exercise* were both significant (*F* (1, 34) = 6.28, *p* = 0.02; *F* (1, 34) = 8.23, *p* = 0.001; Figure 3d).

*Sucrose splash test*

The *ethanol* factor was significant in the length and frequency of grooming behavior (*F* (1, 34) = 10.06, *p* = 0.003; *F* (1, 34) = 18.05, *p* < 0.0001, respectively). Specifically, the EtOH groups spent less time on these behaviors and performed them less frequently, regardless of the exercise condition (Figure 3e,f). Concerning the latency in the commencement of grooming behavior, we found a significant *ethanol x exercise* interaction (*F* (1, 34) = 5.01, *p* = 0.003). Post hoc analysis showed that the EtOH group had higher latency than the EtOH + EX (*p* = 0.04) and the CON groups (*p* = 0.001) (Figure 3g).

*Nest building test*

The quality of the nest improved between the first and second testing sessions (1 h vs. 24 h; *F* (1, 34) = 295.81, *p* < 0.001 The *ethanol* factor was significant (*F* (1, 34) = 3.88, *p* = 0.05), which indicated that animals belonging to this condition built worse quality nests, regardless of the session and exercise training (Figure 3h).

*Three-chamber test*

With regard to the time spent in each compartment (Figure 3i), the *ethanol x exercise* interaction was significant (*F* (1, 34) = 12.13, *p* = 0.001), as was the *chamber x exercise* interaction (*F* (1, 34) = 6.32, *p* = 0.02), which indicated that the exercise groups spent less time in the social compartment. Concerning sniffing behavior (Figure 3j), we observed a significant chamber x ethanol x exercise interaction (*F* (1, 34) = 18.28, *p* < 0.001). Tukey’s post hoc analysis revealed that the CON group performed more social sniffing than the other groups (*p* < 0.001), and the EX group outperformed the EtOH (*p* < 0.001) and EX + EtOH (*p* = 0.01) groups. Finally, treadmill exercise (EX + EtOH) significantly increased the levels of social sniffing relative to the EtOH group (*p* < 0.001).

#### 3.3.2. Spontaneous Alternation and Exploration

*Y-maze*

**Figure 4:**
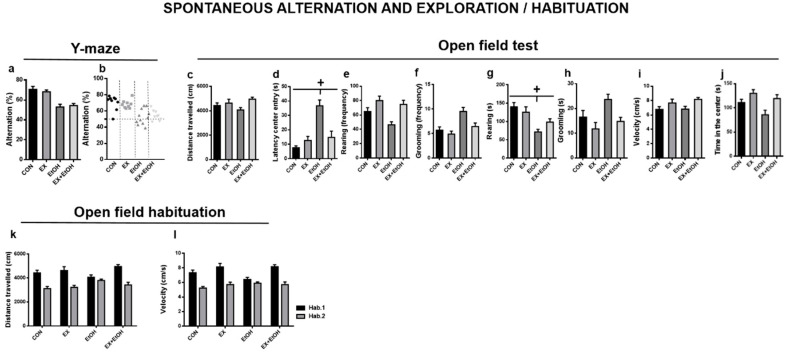
Behavioral performance in Spontaneous alternation and exploration/habituation. (**a**,**b**: Y-maze; **c**–**j**: Open field (exploration); **k**,**l**: Open field (habituation). All data are mean ± SEM, and statistically significant differences were considered when *p* < 0.05. + Represents statistical differences between the EtOH condition compared to other groups.

The factor *ethanol* was significant (*F* (1, 34) = 38.219, *p* < 0.001) indicating that animals in this condition performed a lower number of alternations (Figure 4a). Figure 4b shows the number of mice in each group who performed at chance levels.

*Open field test: Exploration*

Regarding the distance traveled (Figure 4c), the *exercise* factor was significant (*F* (1, 34) = 8.44, *p* < 0.006); the animals who performed exercise travelled longer distance regardless of ethanol condition. In the case of latency in moving to the center of the field (Figure 4d), we found a significant *ethanol x exercise* interaction (*F* (1, 34) = 6.05, *p* = 0.02), and post hoc analyses revealed that the EtOH group showed a higher latency in moving to the center of the field than the other groups (CON: *p* = 0.003, EX: *p* = 0.02, EX + EtOH: *p* = 0.04). In both rearing and grooming frequency variables (Figure 4e,f, respectively), the *ethanol* factor (*F* (1, 34) = 5.02, *p* = 0.03; *F* (1, 34) = 13.80, *p* < 0.001, respectively) and exercise factor (*F* (1, 34) = 16.56, *p* < 0.001; *F* (1, 34) = 6.98, *p* = 0.01, respectively) were significant. In terms of the time spent performing these behaviors (Figure 4g,h), a significant *ethanol x exercise* interaction was found in the rearing variable (*F* (1, 34) = 3.84, *p* = 0.05), and the *exercise* factor was significant in the grooming variable (*F* (1, 34) = 12.89, *p* = 0.001). Post hoc analysis revealed that the EtOH group spent less time performing rearing behavior compared with the CON (*p* < 0.001), EX + EtOH (*p* = 0.04) and EX (*p* = 0.005) groups. Finally, the exercise factor was significant in the velocity and time in the center variables (*F* (1, 34) = 5.02, *p* = 0.03; *F* (1, 34) = 5.02, *p* = 0.03, respectively); exercised mice were thus faster, regardless of ethanol condition, and spent more time in this part of the field (Figure 4i,j, respectively).

*Open field test: Habituation*

We found a significant *habituation session x exercise* interaction (*F* (1, 34) = 11.51, *p* = 0.002) in the distance traveled (Figure 4k); exercised mice traveled long distances in the first habituation session than unexercised mice. However, this effect was not observed in the second session. We also found that the ethanol factor was significant, showing that animals belonging to this condition reduced the distance they traveled, regardless of both exercise and habitation session (*F* (1, 34) = 3.80, *p* = 0.05). With regard to velocity (Figure 4l), we again found a significant *habituation session x exercise* interaction (*F* (1, 34) = 8.23, *p* = 0.005). The exercised mice showed higher velocity distance in the first habituation session than unexercised mice, but this effect was not observed in the second session.

## 4. Discussion

Our EtOH intake protocol induced anxiety-like behaviors in the EPM, lower exploratory activity in the open field, reduced self-care in the sucrose splash test, as well as higher preference for alcohol-related stimuli rather than social interaction. These results are consistent with previous studies that also observed anxiety-like behavioral alterations and reduced exploration which may be involved in perpetuating this pattern of alcohol abuse [33,34]. In our study, the behavioral testing was carried out while the animals were in withdrawal. Ethanol withdrawal is characterized by aversive physical and mental effects [35,36]. Thus, anxiety is a significant contributing factor to the negative reinforcing properties of ethanol during withdrawal and it could be explained in part the results observed in the EPM because it was performed 24 h after the last DID session. These anxiogenic effects associated with acute ethanol withdrawal are evident in both adolescent and adult animals [37]. Thus, adult rats chronically exposed to ethanol and tested during ethanol withdrawal spent less percentage of time on the open arms of the elevated plus-maze (EPM) and made a smaller percentage of entries into the open arms [38]. Increases in anxiety during withdrawal from ethanol have also been observed in other behavioral tests, including a light-dark box, open field, holeboard test, and social interaction test [39,40]. Thus, we also observed that animals from ethanol condition built nests with poorer quality. Several studies have suggested that decreased nest building in small mammals reflects an anhedonic state [41], and this score has also been shown to be sensitive to EtOH withdrawal severity [42].

Interestingly, we observed that mice exposed to BD exhibited increased latency to start grooming when sucrose was squirted on their dorsal coat, suggesting a reduced willingness to self-clean [43]. Regarding grooming behavior, the EtOH group performed more grooming behavior in the open field, but less grooming in the sucrose splash test. It is an interesting point because when rodents are exposed to novelty for short periods (5–10 min) the amount of self-grooming behavior is an index of their fearfulness/anxiety or perceived stress levels [44]. For instance, the rat line Roman High-avoidance (RHA) which is known to be low anxious and fearful respect to the Roman Low-Avoidance (RLA) show much less self-grooming than RLA rats when exposed to a variety of anxiety/fearfulness tests involving novelty, such as the open-field test, and others [45]. Meanwhile, the sucrose splash test is usually employed to assess apathy-related behaviors [46]. Apathy has been defined as a deficit in goal-directed behavior [47] and in rodents, disturbed self-grooming has been proposed as one sort of measurement to assess it [48]. Therefore, when an animal is stressed, it might do not care about the state of its fur and delay its self-cleaning.

On the other hand, based on the previous finding that access to another natural reward can reduce the preference for a drug [49], we observed that, although the time in both chambers (social and non-social) was similar, the EtOH group spent more time sniffing the grid enclosure containing the 20% alcohol bottle instead of the juvenile mouse (Figure 3j). Consistent with the existing literature, social dysfunction and social anxiety-like alterations have been often associated with ethanol intake in animal models and humans [50,51]. In contrast, exercise training (EX + EtOH group) increased the sniffing of the social stimulus and we considered this a very interesting and promising result. To our knowledge, there are no studies on this topic but recent studies have pointed to the possibility that exercise could prevent the development of an addicted phenotype characterized by high levels of drug-seeking behaviors [52]. Moreover, the anxiety reduction observed in this group could also contribute to lower craving levels, preventing relapses, and favoring the approach to more positive rewards. Nevertheless, further studies are necessary to examine these possibilities, as well as the employment of other behavioral avenues, such as ethanol self-administration protocols.

In contrast, spontaneous alternation, as a measure of spatial working memory, was impaired after our DID procedure, but it was not rescued by exercise training. Additionally, in the Open field habituation, we found that animals belonging to ethanol condition reduced the distance traveled regardless of both exercise and habitation session. Therefore, we cannot conclude that the EtOH group exhibited reduced habituation to a spatial context. These results seem to suggest that treadmill exercise training might induce more positive effects in emotionality and exploration-related domains than in cognitive areas such as memory. Several brain neurotransmitter systems, including serotonergic [53], GABAergic [54], and glutamatergic systems [55] are involved in controlling anxiety-related behaviors and it has been demonstrated that modulations in serotonergic, noradrenergic, and GABAergic neurotransmission may be involved in anxiolytic effects of exercise [56]. On the other hand, in our study, treadmill exercise did not reduce binge alcohol consumption over time (Figure 2a), which has also been observed in other studies in which voluntary wheel running seemed to have a more positive effect on other patterns of alcohol drinking [57]. We also want to clarify that although the animals were increasing their alcohol intake over the weeks, we did not observe until the fifth-week levels considered as binge-like drinking/intoxication (typically 80–100 mg/dl) (Figure 2b) which could have influenced in the observed behavioral results.

Moreover, and in contrast to our expectations, the exercise protocol used in this study produced limited behavioral changes compared to the sedentary CON group. This result could be due to methodological variables such as exercise duration or intensity. Thus, most studies that have applied this sort of exercise usually employed longer protocols per day and for more weeks [58,59]. Nonetheless, despite the length of our exercise protocol, it is noteworthy that we found a positive behavioral effect in some measurements when it concurred with ethanol intake. Thus, exercise-related aspects such as the level, type, or timing of exposure must be considered when this type of non-pharmacological intervention is designed, given that it activates many of the same circuits as drugs of abuse. Moreover, it is difficult to discriminate whether the behavioral effects observed in our study are due to the effects of withdrawal, or are they persistent consequences of ethanol exposure.

In conclusion, our results suggest that a moderate protocol of treadmill exercise could be a valuable strategy to counteract some behavioral alterations associated with ethanol intake during adolescence and early adulthood. Specifically, we observed that BD mice with daily treadmill exercise displayed fewer anxiolytic-related behaviors, more exploratory activity, better body care efficiency, and reduced preference for alcohol odor when another rewarding stimulus was present. In contrast, other registered measures were not improved by treadmill exercises such as working memory or habituation learning. Therefore, it is critical to identify exercise conditions that produce beneficial effects, as this non-pharmacological intervention is considered a potential treatment for addiction and other psychiatric disorders. For instance, it has been also described that the treadmill exercise effects may depend on the circadian rhythm of the mice, so it has been found that the benefits were higher in mice that exercised during the day or in the evening than in mice that exercised at dawn [60]. In our case, animals were under light-dark inversed cycle (red light on at 9:00 h—red light off at 21:00 h) the point at which the animals started to become more active and often displayed better cognitive performance [61,62]. Animals from EX+ EtOH and EX groups were trained within the same circadian period (12 h dark cycle), so this variable did not impact the benefits of treadmill exercise on cognition.

## Supplementary Materials

The following are available online at https://www.mdpi.com/2076-3425/10/9/576/s1, Supplementary methods for the behavioral assessment and supplementary statistical analysis.
