# Supplementary material for: Treadmill Exercise Buffers Behavioral Alterations Related to Ethanol Binge-Drinking in Adolescent Mice
Source: Brain Sci. 2020 Aug 20;10(9):576. doi: 10.3390/brainsci10090576 (PMC7563508; doi:10.3390/brainsci10090576)
Supplement: Supplementary file 1 [file brainsci-10-00576-s001.pdf]

SUPPLEMENTARY MATERIAL

SUPPLEMENTARY METHODS FOR THE BEHAVIORAL ASSESSMENT:

EPM

Elevated plus maze apparatus was raised 55 cm above the floor and consisted of two open arms (37 x 5 cm) and two closed arms (37 x 5 cm surrounded by opaque walls 16.5 cm high) connected by a central platform (5 x 5 cm).

Y maze

Rodents tend to explore the least recently visited arm alternating the visits between the three arms. Hence, working memory is necessary to maintain an ongoing record of most recently visited arms, and continuously update such record [1]. The maze consisted of three equally sized arms (30 x 14 cm each), with opaque walls (33 cm high).

Open field

Mice were allowed to explore a squared open field (42 x 42 cm; 40 cm high) with a central area considered as a 20 x 20 cm square.

Nest building test

The scoring system for assessing nest quality was based on Deacon's standardized scale [2]: 1 = the Nestlet is mostly untouched (<10% shredded); 2 = part of the Nestlet is torn, but most of it remains intact (10% - 50% shredded); 3 = most of the Nestlet is torn, but more than 10% remains intact (50% - 90% shredded), or the material is scattered in the cage without forming an identifiable nest site. If the Nestlet was completely torn (> 90% shredded) and gathered into a nest, the nest was conceptually divided into four equally sized quadrants. Each nest quadrant was then given a '4' score when its wall was mostly flat or a '5' score when its wall was high (i.e., mostly covering the mouse's body height when the animal is curled up on its side), and the scores of all four quadrants were averaged. Thus, scores from 4 to 5 ranged from a functional but completely flat nest (= 4) to the 'perfect' crater-like nest with walls that covered the mouse all around its circumference (= 5).

Three chamber test session

The apparatus structure is shown in Supplementary figure 1. As it can be observed, rewarding stimulus were placed in the opposite chambers and the tested animal in the central chamber.

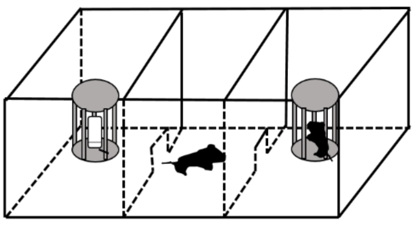

Apparatus was made up by Panlab, Harvard apparatus (box: 60 x 42 x 22 cm; compartment: 20 x 42 x 22 cm). In the test session (10 min), the mice could choose to explore a juvenile mouse (3-4 weeks old) placed in one grid enclosure (3 mm x 7.4 mm) or a bottle with 20% of alcohol in tap water (20 mL) in the opposite grid enclosure. As we mentioned in the manuscript, time spent in each chamber, as well as the time performing sniffing behaviour was registered. The juvenile mouse and the alcohol was replaced for new ones after each session.

SUPPLEMENTARY STATISTICAL ANALYSIS:

Those behavioral variables registered without significant differences between groups will be shown below. Two-way ANOVAs, in which exercise (exercise/unexercised) and ethanol (ethanol/no ethanol) were the two factors with two levels each, were carried out:

EPM

At the beginning of the trial, the mouse was placed in the centre of the EPM. Hence, as a measure of looking for protection, we registered the first latency to enter into a closed arm. As it can be seen in the Figure, we did not observe a significant interaction *ethanol x exercise* (F (1, 34) = 0.24, p = 0.63) nor the main effects *ethanol* (F (1, 34) = 0.007, p = 0.93) and *exercise* (F (1, 34) = 2.41, p = 0.13).

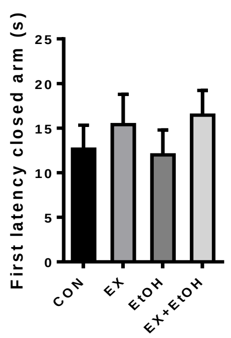

Y-maze test

Regarding the number of entries, the factors *ethanol* (F (1, 34) = 3.74, p = 0.06) and *exercise* (F (1, 34) = 0.21, p = 0.65) were not significant, nor the interaction between them (F (1, 34) = 0.21, p = 0.65). Finally, the main factors (*ethanol*: F (1, 34) = 2.99, p = 0.09; *exercise*: F (1, 34) = 0.39, p = 0.54) and the interaction *ethanol x exercise* (F (1, 34) = 0.33, p = 0.57) were not significant in the variable percentage of perseverations.

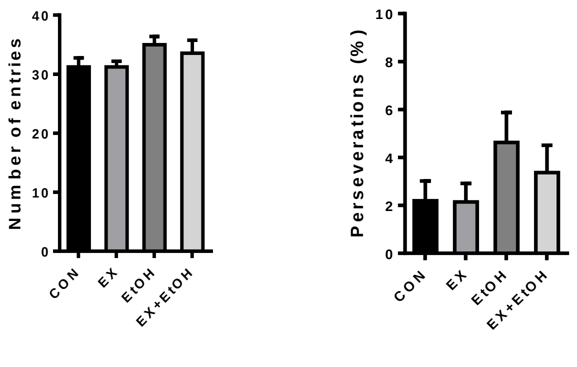

REFERENCE

[1] Wietrzych, M.; Meziane, H.; Sutter, A.; Ghyselinck, N.; Chapman, P.F.; Chambon, P.; Krezel, W. Working memory deficits in retinoid X receptor gamma-deficient mice. Learn. Mem. 2005, 12, 318-326.

[2] Deacon, R.M.J. Assessing nest building in mice. Nat. Protoc. 2006, 1, 1117-111.
